# Does the Earth's Magnetic Field Serve as a Reference for Alignment of the Honeybee Waggle Dance?

**DOI:** 10.1371/journal.pone.0115665

**Published:** 2014-12-26

**Authors:** Veronika Lambinet, Michael E. Hayden, Marco Bieri, Gerhard Gries

**Affiliations:** 1 Department of Biology, Simon Fraser University, Burnaby, British Columbia, Canada; 2 Department of Physics, Simon Fraser University, Burnaby, British Columbia, Canada; University of North Carolina, Greensboro, United States of America

## Abstract

The honeybee (*Apis mellifera*) waggle dance, which is performed inside the hive by forager bees, informs hive mates about a potent food source, and recruits them to its location. It consists of a repeated figure-8 pattern: two oppositely directed turns interspersed by a short straight segment, the “waggle run”. The waggle run consists of a single stride emphasized by lateral waggling motions of the abdomen. Directional information pointing to a food source relative to the sun's azimuth is encoded in the angle between the waggle run line and a reference line, which is generally thought to be established by gravity. Yet, there is tantalizing evidence that the local (ambient) geomagnetic field (LGMF) could play a role. We tested the effect of the LGMF on the recruitment success of forager bees by placing observation hives inside large Helmholtz coils, and then either reducing the LGMF to 2% or shifting its apparent declination. Neither of these treatments reduced the number of nest mates that waggle dancing forager bees recruited to a feeding station located 200 m north of the hive. These results indicate that the LGMF does not act as the reference for the alignment of waggle-dancing bees.

## Introduction

The waggle dance of the honeybee, *Apis mellifera*, is performed by a forager bee inside the hive and informs nest mates about the existence and location of a rich food source [1]. It is undoubtedly one of the most sophisticated means of information transfer amongst insects and probably the entire animal kingdom.

**Figure 1:**
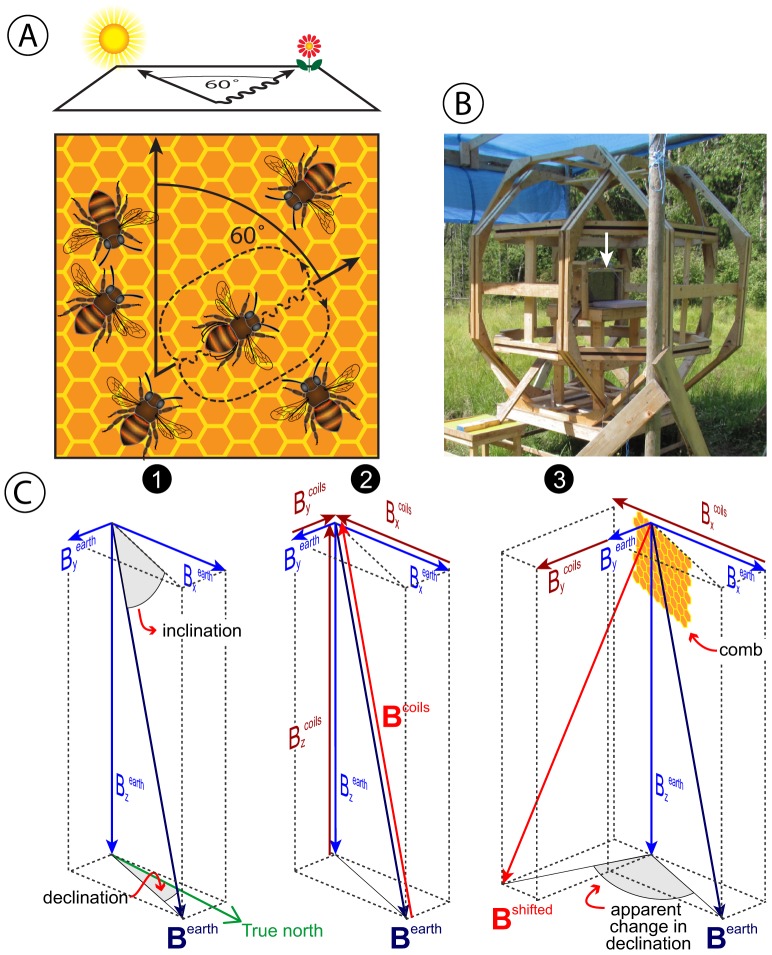
Experimental design to test the effect of the Earth's magnetic field on the recruitment success of waggle-dancing honeybees. (A) bottom: waggle dancing honeybee describing a figure 8 on the vertical comb in a hive; top: the angle of the waggle run relative to vertical correlates with the angle between the target food source and the azimuth of the sun (angular direction); (B) observation hive (see arrow) inside a triaxial Helmholtz coil system capable of cancelling or arbitrarily modifying the local geomagnetic field; (C1) components (blue vectors) of the local (unmodified) geomagnetic field (B^earth^); (C2 & C3) manipulation of field components (red vectors) to cancel the local geomagnetic field (C2) or to rotate its declination to the East (C3).

The waggle dancing bee describes a figure 8 on a vertical comb in the hive (Fig. 1A) [1]. The straight portion of her dance, known as the “waggle-run”, consists of a single stride [2] emphasized by lateral waggling motions of the abdomen at 12–15 Hz. The angle at which the waggle run is performed on the vertical comb correlates with the angle between the target food source and the azimuth of the sun (angular direction) or that of sun-linked patterns of polarized skylight (Fig. 1A) [1], [3]. The distance to the food source is encoded in the number of sound pulses generated during the waggle run [4], with more pulses conveying a more distant food source. Following each waggle run, the bee alternately turns left or right and returns to her starting point, describing a figure 8 in the process. She may repeat this procedure many times. The hypothesis that the waggle dancing bee recruits hive mates to a food source has been confirmed by radar-tracking the flight of bees that attended a waggle dance [5].

The directional information pointing to the location of a food source is encoded in the angle between two lines, the waggle run line and a reference line. Gravity was implicated as the natural basis for this reference line following the discovery of a gravity-sensing organ, consisting of paired hairs at the joints between the head and thorax, and the thorax and abdomen [6]. This hypothesis is supported by findings that bees with experimentally impaired gravity receptors failed to perform meaningful waggle dances. It is conceivable however, that the impairment procedure inhibited the bees' ability to waggle dance properly, and that instead the local (ambient) geomagnetic field (LGMF) serves as the reference line. Like gravity, the LGMF is uniform and stable over long distances.

The ability of bees to detect the LGMF has been reported many times; a few examples are highlighted here. Both the orientation of a vertical comb with respect to the LGMF and the intensity of the LGMF affect the ability of bees to describe food locations during their waggle dances, and alter the bees' dance behavior [7], [8]. When bees are forced to waggle dance on a horizontal comb, they align their dance primarily N-S or E-W irrespective of the location of the food source [8], particularly when the intensity of the LGMF is experimentally increased by a factor of 10. On the other hand, bees dancing on horizontal combs fail to align their dance N-S or E-W when the intensity of the LGMF is reduced by 96% [8].

Studies of the apparent magnetoreceptivity of bees revealed their sensitivity to changes in the magnetic field [9], their sensitivity to the direction of the magnetic field [10], and their insensitivity to alternating magnetic fields [11]. Nevertheless, the nature of the receptor that is involved, the manner in which it functions, and its location are unknown or controversial [12]–[18].

In light of the paramount importance of honeybees as crop pollinators [19], [20], it is essential that we understand their in-hive communication system, which ultimately recruits nest mates to crops. We need to demonstrate unequivocally whether the directions established by the Earth's gravitational or magnetic fields serve as a reference line for waggle dancing bees. Because honeybees are sensitive to the LGMF [9] and may sense residual fields, rigorous LGMF manipulations should include both a reduction in field strength and a shift of its declination around the hive. This has not yet been done. Moreover, previous studies that altered the strength of the LGMF to test its effect on in-hive communication of bees report misdirections (“Missweisungen” [7], [8]) during waggle dances as the experimental response criterion rather than the all important success of a dancing bee to recruit nest mates to a food source.

Given the current state of knowledge, the directions established by both the Earth's gravitational and magnetic fields are plausible reference lines for the bees' waggle dance. Experimental tests of either field as the basis for a reference line require the ability to manipulate or eliminate the relevant field strength and/or direction. As it is difficult to modify gravity without affecting bee behavior, we focused our studies on modulating the LGMF in the vicinity of hives. We carried out experiments in which we suppressed (reduced to 2%), or rotated the declination of the magnetic field around a hive, and assessed the subsequent success of waggle dancing bees to recruit nest mates to a feeding station.

## Results

A set of six large current-carrying coils arranged to form three orthogonal Helmholtz pairs (Fig. 1B) allowed us to arbitrarily control the ambient magnetic field around a single-frame observation hive containing up to 2000 individually marked bees. During 2-h treatment sessions, but not 2-h control sessions, we reduced the measured intensity of the ambient magnetic field in the hive to <2% of the LGMF (residual fields: 0.8 µT ±0.5 µT; stability limited by the influence of environmental temperature changes on the magnetometer and current sources).

**Figure 2:**
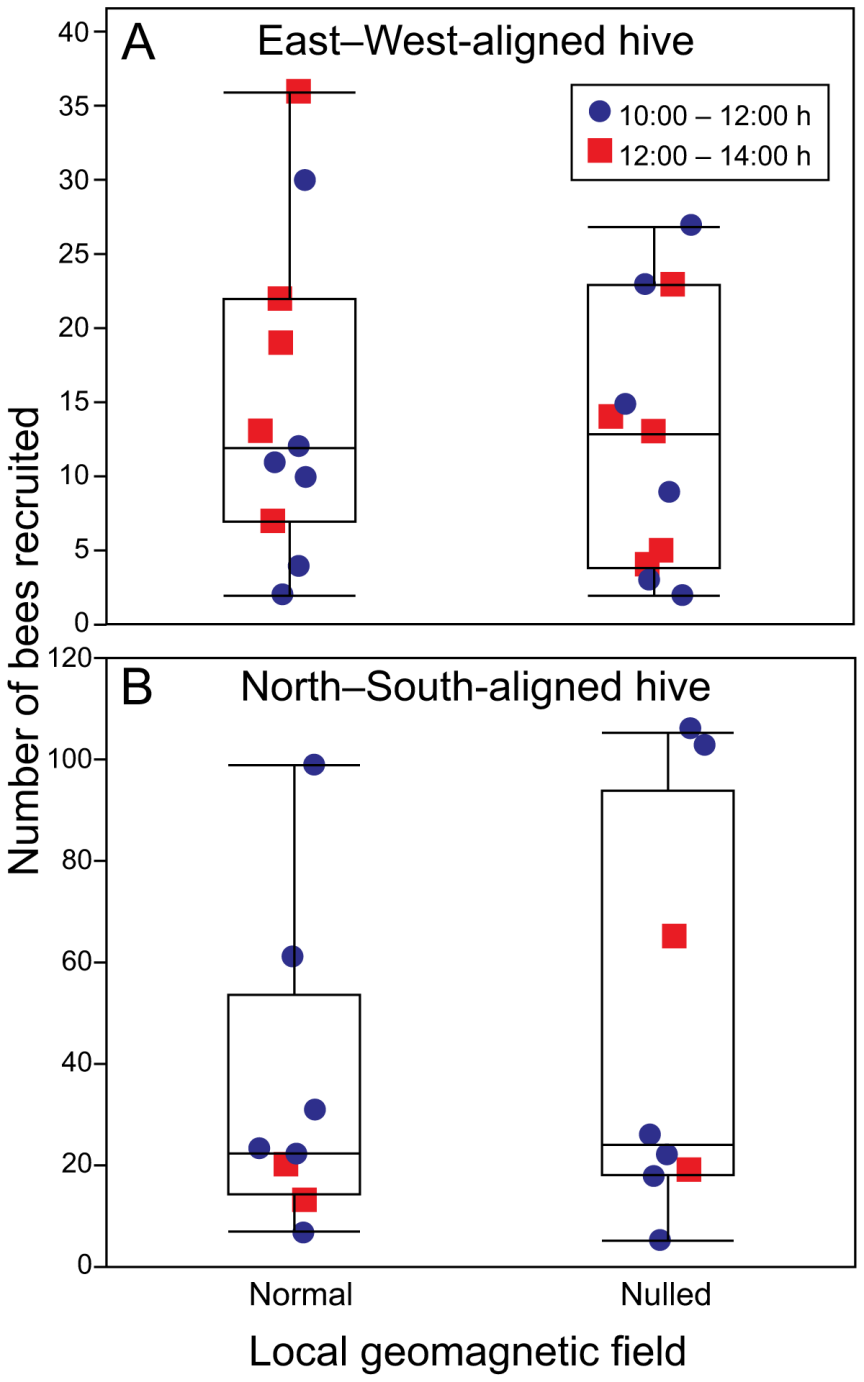
Effect of suppressing the ambient magnetic field on the recruitment success of waggle-dancing bees. Boxplots show the mean, median lower and upper quartiles, and ± whiskers (minimum/maximum data points) of the number of honeybee nest mates recruited in morning and afternoon sessions to a feeding station in the presence (control session) or absence (treatment session) of the ambient magnetic field (see Fig. 1B, C2). The presence or absence of the ambient magnetic field had no effect on the number of recruits irrespective of the alignment of the observation hive (East–West: linear mixed effect model analysis; F_1,10_ = 0.9548, p = 0.35; North–South: linear mixed effect model analysis; F_1,7_ = 0.9660, p = 0.3).

In experiment 1 (East–West alignment of hive) and experiment 2 (North–South alignment of hive), the number of nest mates that forager bees recruited to a feeding station located 200 m north of the hive did not differ between treatment and control sessions [experiment 1: F_1,10_ = 0.9548, p = 0.35 (Fig. 2A); experiment 2: F_1,7_ = 0.9660, p = 0.36 (Fig. 2B)], indicating that the LGMF had no effect on the ability of forager bees to recruit nest mates to a food source.

**Figure 3:**
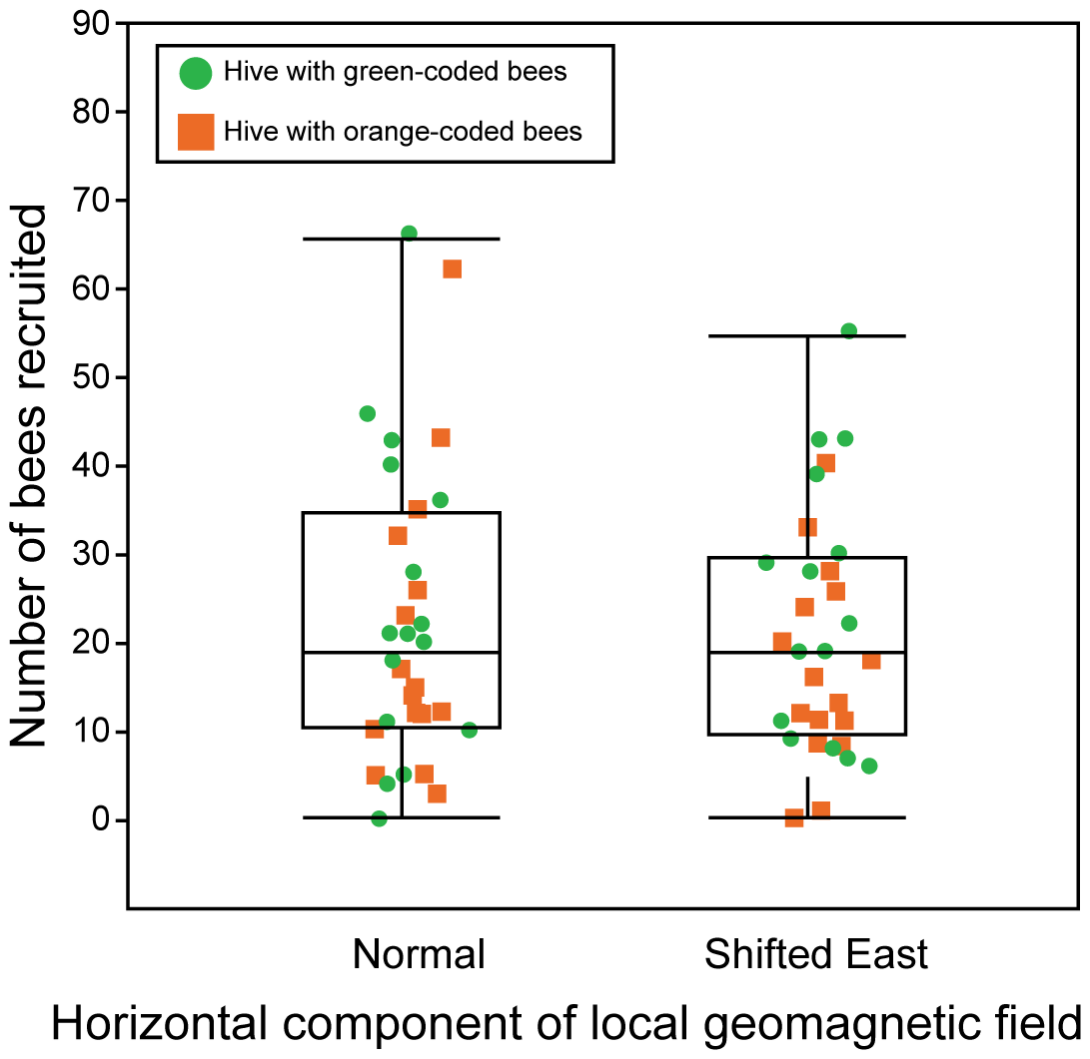
Effect of shifting the declination of the ambient magnetic field to the East on the recruitment success of waggle-dancing bees. Boxplots show the mean, median, lower and upper quartiles, and ± whiskers (minimum/maximum data points) of the number of honeybee nest mates recruited to a feeding station when the hive was exposed to either the ambient magnetic field (control session) or to the magnetic field with its declination shifted East (treatment session) (see Fig. 1, C3). In each replicate (N = 15), treatment and control sessions were run concurrently using a single-frame observation hive with green- or orange-coded bees in each of two separate but identical Helmholtz coil systems (one of which is shown in Fig. 1B). The number of nest mates recruited from hives in treatment or control sessions did not differ (linear mixed effect model analysis; F_1,14_ = 0.0274, p = 0.87).

In experiment 3, we deployed a single-frame observation hive with orange- or green-coded bees in each of two separate Helmholtz coil systems; during 2-h treatment sessions, but not concurrently-run 2-h control sessions, we rotated the declination of the ambient magnetic field toward the East (Fig. 1, C3), on average by 84°±4°, while maintaining its intensity constant within <3% (±1.4 µT). This manipulation essentially achieves a rotation of the horizontal component of the magnetic field. In experiment 3, forager bees recruited similar numbers of nest mates to the feeding station during treatment and control sessions [F_1,14_ = 0.0274, p = 0.87 (Fig. 3)], again indicating that the direction of the LGMF field had no effect on the ability of forager bees to recruit nest mates to a food source.

## Discussion

Our data show that suppressing the LGMF to 2% of its nominal value has no effect on the number of nest mates that forager bees recruit to a feeding station, irrespective of hive alignment North–South or East–West. Furthermore, our data show that rotating the declination of the field from North to East had no effect on the number of recruits to a feeding station. Combined, these results support the conclusion that the LGMF is not likely being used by honeybees as a reference line during the waggle dance. With the LGMF experimentally eliminated as the possible reference, the direction of the Earth's gravitational field is the obvious alternative reference line.

The gravitational field always points to the center of the Earth and would make a highly reliable reference line for the waggle run. It was already implicated as a potential reference for the waggle-run alignment when Lindauer and Nedel [6] discovered a gravity receptor in bees, and discussed its potential role in the context of their waggle dance. The perfectly vertical honeycombs that bees build in their nest may facilitate optimal detection and reading of this reference during information conveyance in the waggle dance.

The suppression of the LGMF by >98% in experiments 1 and 2 of our study was likely sufficient to reveal any potential effect of the LGMF on the recruitment success of waggle-dancing bees. Even a 96-% suppression of the LGFM was previously shown to have a distinct effect on the waggle dance behavior of bees [7]. In a 96-% suppressed LGMF bees exhibit smaller communication inaccuracies (“Missweissungen”) on vertical combs, and align their dance randomly (instead of N-S or E-W) on horizontal combs [8]. Even if bees sensed the residual magnetic field in experiments 1 and 2 [9] and thus prevented us from detecting an LGMF-treatment effect, the results of experiment 3 indicate that the LGMF is not essential for communication conveyance of waggle dancing bees. In experiment 3, we shifted the declination of LGMF without affecting the number of nest mates that waggle dancing bees recruited to a feeding station (Fig. 3). These results provide strong evidence that the LGMF is not the reference line for the waggle dance alignment of bees.

The ability of bees to sense magnetic fields [7]–[10], [21]–[22] is well accepted but the biological significance of this ability is poorly understood. Its single known function is in the context of comb alignment. Bees building combs in a new hive orient them according to the comb alignment in their parent hive by making reference to the LGMF [23]. Another factor that is poorly understood is the biological significance of the sensitivity of the bees' magnetoreceptor, which can sense changes as small as 0.6% of the LGMF [9]. If the magnetoreceptor plays a significant role in the bees' everyday life, the ever increasing magnetic noise pollution of the industrial world could interfere with its optimal functioning and could contribute to the bees colony collapse disorder [18].

In conclusion, we have tested a potential role of the LGMF in the waggle dance language of honeybees. Neither cancelling the ambient magnetic field nor shifting its declination from North to East had any measurable effect on the recruitment success of waggle-dancing forager bees. These results likely eliminate the LGMF as a reference for the waggle-run alignment of a dancing bee that attempts to inform her hive mates about a food source. These results also implicate, yet again [6]–[8], gravity as the more plausible reference line, which together with the waggle-run line of the dancing bee appears to form the angle that encodes the direction to a food source. Considerable speculation has been devoted to the manner in which hive mates read the waggle-run line of the dancing bee [24]–[32] but the definitive answer has yet to be found.

## Material and Methods

### Experimental location

We ran all experiments on a property near Sicamous, British Colombia, Canada (50°52N, 118°56W) where no power lines or roads interfered with the measured LGMF. The local magnetic field intensity was 55 µT±1.3 µT which is consistent with expectations for this location (intensity: 56 µT; inclination: 72°; declination: 17° East [33]).

### Manipulation of ambient magnetic fields

We custom-built two triaxial Helmholtz coil systems (Fig. 1B) to control the ambient magnetic field around a single-frame observation hive (51 cm wide ×29 cm high ×13 cm deep). We assembled each of the six (regular) octagonal coils from wood studs (3.8×8.9 cm), with a groove (2 cm wide ×1.5 cm deep) cut into one surface to accommodate 21 turns of insulated solid 14 AWG wire. With respect to the hive orientation, the coils producing the vertical-, perpendicular-, and horizontal in-plane components of the applied magnetic field had minimum outer diameters of 1.70 m, 1.85 m, and 2.10 m, respectively. Under typical operating conditions the fields produced by these coils are uniform over the hive volume at the level of 1% or better.

We altered the ambient magnetic field around the observation hive by independently controlling the current in each of the three pairs of coils using Hewlett Packard 6002A power supplies (Hewlett-Packard Company, Palo Alto CA, USA). We deployed a Honeywell model HMC 2003 three-axis magnetic sensor (Honeywell International Inc., Plymouth, MN, USA) to monitor the magnetic field at the hive; we acquired data using a DATAQ Instruments model DI-149 data acquisition system and processed it using WinDaq/XL software (DATAQ Instruments, Akron, Ohio, USA).

### Preparation of the observation hive

We placed a single-frame Plexiglas observation hive containing up to 2000 individually marked bees in the center of the Helmholtz coil system. Marking bees allowed us to recognize them as recruits in experiments and to distinguish them from other bees on the property. To be able to mark young bees (<24-h-old) that were not yet able to fly and sting, we kept a frame with bee larvae and pupae in the observation hive for 19 days and then transferred it to an incubator (Narco, Model 310, San Diego, CA, USA). We checked the incubator every 24 h and collected emergent bees. In experiments 1 and 2 (2011), we marked bees individually with colored-number tags (Wienold Imkereibedarf, Lauterbach, Germany). In experiment 3 (2012; North-South alignment of the hive) during which we concurrently deployed two observation hives, we again marked bees individually but coded them with an orange or green dot (Opaque Paint Markers; Elmer's Products Inc., Toronto, ON, Canada) as an indicator of the hive from which they originated.

During experiments, we excluded visual cues inside the hive by (*i*) placing thick pieces of Styrofoam over the transparent walls of the hive, (*ii*) using duct tape to seal all gaps between the Styrofoam and the wood frame of the hive, and (*iii*) taping paper tubes over the hive entrance to block direct sunlight. This was done because inhomogeneous comb illuminations can have an adverse effect on the waggle dance [1]. Thus, instead of using illuminated hives and recording the accuracy of waggle dance alignments (or the degree of misdirections [8]) as the experimental treatment effect, we measured the success of waggle dancing bees to recruit nest mates to a feeding station 200 m away from the hive.

### Training of bees to visit a feeding station

We trained bees to visit a foraging station consisting of a table (45 cm×45 cm×60 cm high) with a yellow plastic cover as a distinct visual cue. At the foraging station we offered bees a watch glass (2.5 cm diameter) filled with anise-scented sugar water or an empty honeycomb (3 cm×6 cm) filled with diluted honey. Once bees had learned to locate and revisit the foraging station near the observation hive, we moved the station in multiple steps to its final destination 200 m north of the observation hive. We trained three bees from each hive to forage at the station (henceforth “foragers”) and allowed them to recruit other nest mates (henceforth “recruits”) to the station.

### Experiments 1 and 2 (August to September, 2011): Effect of suppressed ambient magnetic field on the recruitment success of bees

We suppressed the LGMF at the hive by applying opposing fields directed along the vertical, horizontal, and perpendicular directions (with respect to the hive) as shown in Fig. 1, C2. In experiment 1 (3–29 August 2011), we aligned the plane of the observation hive perpendicular (East–West) to the horizontal component of the LGMF; in experiment 2 (14–28 September 2011), we aligned the plane of the observation hive parallel (North–South) to the horizontal component of the LGMF. We tested the same hive in both experiments, but separated the experiments by two weeks so that the bees could adapt to the new magnetic field alignment. Prior to the first experiment, the plane of the observation hive had been in East–West alignment for 2.5 weeks.

Each replicate of the experiment involved a treatment and a control session. During the 2-h treatment session, but not the 2-h control session, we cancelled the ambient magnetic field around the colony. We alternated the order of treatment and control sessions daily. Each replicate commenced after one of the three foragers arrived at the feeding station. We photographed every bee at the station to document the time and number of visits for each bee. We allowed foragers to return to the colony, but aspirated recruits that landed on the station in a glass jar so that they could not recruit other nest mates.

### Experiment 3 (September 2012): Effect of shifting the magnetic declination on the recruitment success of bees

We shifted the declination of the magnetic field at the location of the hive from North to East, by applying currents to the Helmholtz coils (Fig. 1, C3). The change was made so as to maintain the intensity and the inclination of the field constant. The experimental design for testing the recruitment success of bees consisted of a single-frame, magnetic North–South-aligned observation hive, in each of two separate concurrently run Helmholtz coil systems. Each experimental replicate had a 2-h treatment and a 2-h control session. During the treatment session, but not the control session, we shifted the ambient magnetic declination in the vicinity of the hive toward the East (on average, by 84°±4°). During each of two experimental replicates per day, we assigned one hive to a treatment session and the other to a control session, alternating the sequence of assignments of treatment or control session for each hive between days. We took day- and time-of-day-stamped photographs during sessions to document recruits at the feeding station, and to compare the number of recruits during treatment and control sessions. These photographs also enabled us to determine the exact time the first forager bee arrived at the station, at which time we started the experiment.

### Data analyses

We analyzed data from experiments 1 and 2 using a linear mixed effect model of the form: *Log (Recruits)  =  Treatment + Date(R)*. The *Treatment* term is a fixed effect representing the reduction in the number of recruited nest mates for treated hives, while *Date(R)* is a random effect representing the day-to-day variability in the number of recruits. We used a log transformation of the number of recruits to normalize the data, and to be able to report the *Treatment* effect as a direct estimate of the Log (Recruits) ratio, where the biological meaning of a recruitment ratio is easy to interpret.

We analyzed data from experiment 3 using a similar linear mixed effect model, but included additional random effects to account for modifications of the experimental design: Log(Recruits)  =  Treatment + Date(R) + Hive(R) + Date*Hive(R) + Date*Time of Day(R). Here *Hive(R)* accounts for differences between hives, Date*Hive(R) accounts for differences between hives on a given day, and Date*Time of Day(R) accounts for potential differences in the number of recruits between morning and afternoon sessions. By accounting for these additional sources of variability we reduced the residual error by more than a factor of two, thus increasing the statistical power to detect the main *Treatment* effect (reduction in recruited nest mates). All analyses were executed using JMP 9.0.2 (JMP, Version *9.0.2*. SAS Institute Inc., Cary, NC, 1989–2007).

## References

[1] Von Frisch K (1968) The dance language and orientation of bees. Harvard University Press.

[2] Von Frisch K (1948) Gelöste und ungelöste Rätsel der Bienensprache. Naturwissenschaften 35:38–43 Available: 10.1007/BF00626789 [doi].

[3] Von Frisch K (1949) Die Polarisation des Himmelslichtes als orientierender Faktor bei den Tänzen der Bienen. Experientia 5:142–148 Available: 10.1007/BF02174424 [doi]. 18126348

[4] Wenner A (1962) Sound production during the waggle dance of the honey bee. Anim Behav 10:79–95 Available: 10.1016/0003-3472(62)90135-5 [doi].

[5] Riley JR, Greggers U, Smith AD, Reynolds DR, Menzel R (2005) The flight paths of honeybees recruited by the waggle dance. Nature 435:205–207 Available: 10.1038/nature03526 [doi]. 15889092

[6] Lindauer M, Nedel O (1959) Ein Schweresinneorgan der Honigbiene. Z vergl Physiol 42:334–364 Available: 10.1007/BF00298125 [doi].

[7] Lindauer M, Martin H (1968) Die Schwereorientierung der Bienen unter dem Einfluß des Erdmagnetfeldes. Z vergl Physiol 60:219–243 Available: 10.1007/BF00298600 [doi].

[8] Martin H, Lindauer M (1977) Der Einfluß des Erdmagnetfeldes auf die Schwereorientierung der Honigbiene (*Apis mellifica*). J Comp Physiol A 122:145–187 Available: 10.1007/BF00611888 [doi].

[9] Walker MM, Bitterman ME (1989) Honeybees can be trained to respond to very small changes in geomagnetic field intensity. J Exp Biol 149:489–494.

[10] Kirschvink JL, Kobayashi-Kirschvink A (1991) Is Geomagnetic Sensitivity Real? Replication of the Walker-Bitterman Magnetic Conditioning Experiment in Honey Bees. Amer Zool 31:169–185 Available: 10.1093/icb/31.1.169 [doi].

[11] Kirschvink JL, Padmanabha S, Boyce C, Oglesby J (1997) Measurement of the threshold sensitivity of honeybees to weak, extremely low-frequency magnetic fields. J Exp Biol 200:1363–1368. 9319256 10.1242/jeb.200.9.1363

[12] Gould JL, Kirschvink JL, Deffeyes KS (1978) Bees Have Magnetic Remanence. Sience 201:1026–1028 Available: 10.1126/science.201.4360.1026 [doi]. 17743635

[13] Desoil M, Gillis P, Gossuin Y, Pankhurst QA, Hautot D (2005) Definitive identification of magnetite nanoparticles in the abdomen of the honeybee *Apis mellifera* . J Phys Conf Ser 17:45–49 Available: 10.1088/1742-6596/17/1/007 [doi].

[14] Hsu C, Li C (1994) Magnetoreception in honeybees. Science 265:95–97 Available: 10.1126/science.265.5168.95 [doi]. 17774695

[15] Hsu C-Y, Ko F-Y, Li C-W, Fann K, Lue J-T (2007) Magnetoreception system in honeybees (*Apis mellifera*). PLOS ONE 2:e395 Available: 10.1371/journal.pone.0000395 [doi]. 17460762 PMC1851986

[16] Kuterbach DA, Walcott B (1986) Iron-containing cells in the honey bee (*Apis mellifera*) I. Adult morphology and physiology. J Exp Biol 126:375–387 Available: 10.1126/science.218.4573.695 [doi]. 3805998

[17] Walker MM (2008) A model for encoding of magnetic field intensity by magnetite-based magnetoreceptor cells. J Theor Biol 250:85–91 Available: 10.1016/j.jtbi.2007.09.030 [doi]. 18028964

[18] Válková T, Vácha M (2012) How do honeybees use their magnetic compass? Can they see the North? Bull Entomol Res 102:461–467 Available: 10.1017/S0007485311000824 [doi]. 22313997

[19] Breeze TD, Bailey AP, Balcombe KG, Potts SG (2011) Pollination services in the UK: How important are honeybees? Agric Ecosyst Environ 142:137–143 Available: 10.1016/j.agee.2011.03.020 [doi].

[20] Klein AM, Vaissière BE, Cane JH, Steffan-Dewenter I, Cunningham SA, et al (2014) Importance of pollinators in changing landscapes for world crops. Proc R Soc B 274:303–313 Available: 10.1098/rspb.2006.3721 [doi]. PMC1702377 17164193

[21] Walker M, Bitterman M (1989) Attached magnets impair magnetic field discrimination by honeybees. J Exp Biol 141:447–451.

[22] Walker MM, Baird DL, Bitterman ME (1989) Failure of stationary but not of flying honeybees (*Apis mellifera*) to respond to magnetic field stimuli. J Comp Psychol 103:62–69 Available: 10.1037/0735-7036.103.1.62 [doi].

[23] De Jong D (1982) Orientation of comb building by honeybees. J Comp Physiol A 147:495–501 Available: 10.1007/BF00612015 [doi].

[24] Dreller C, Kirchner WH (1993) How honeybees perceive the information of the dance language. Naturwissenschaften 80:319–321 Available: 10.1007/BF01141904 [doi].

[25] Michelsen A (1993) The transfer of information in the dance language of honeybees: progress and problems. J Comp Physiol A 173:135–141 Available: 10.1007/BF00192972 [doi].

[26] Michelsen A, Andersen B (1992) How honeybees perceive communication dances, studied by means of a mechanical model. Behav Ecol Sociobiol 30:143–150 Available: 10.1007/BF00166696 [doi].

[27] Tanner DA, Visscher PK (2008) Do honey bees average directions in the waggle dance to determine a flight direction? Behav Ecol Sociobiol 62:1891–1898 Available: 10.1007/s00265-008-0619-z [doi].

[28] Tanner DA, Visscher K (2009) Does the body orientation of waggle dance followers affect the accuracy of recruitment? Apidologie 40:55–62 doi: doi:10.1051/apido. Available: 10.1051/apido [doi].

[29] Thom C, Gilley DC, Hooper J, Esch HE (2007) The scent of the waggle dance. PLOS Biol 5:e228 Available: 10.1371/journal.pbio.0050228 [doi]. 17713987 PMC1994260

[30] Tsujiuchi S, Sivan-Loukianova E, Eberl DF, Kitagawa Y, Kadowaki T (2007) Dynamic range compression in the honey bee auditory system toward waggle dance sounds. PLOS ONE 2:e234 Available: http://dx.doi.org/0.1371/journal.pone.0000234 [doi]. 17311102 10.1371/journal.pone.0000234 PMC1794319

[31] Nieh JC, Tautz J (2000) Behaviour-locked signal analysis reveals weak 200–300 Hz comb vibrations during the honeybee waggle dance. J Exp Biol 203:1573–1579. 10769219 10.1242/jeb.203.10.1573

[32] Rohrseitz K, Tautz J (1999) Honey bee dance communication: waggle run direction coded in antennal contacts? J Comp Physiol A 184:463–470 Available: 10.1007/s003590050346 [doi].

[33] Finlay CC, Maus S, Beggan CD, Bondar TN, Chambodut A, et al (2010) International Geomagnetic Reference Field: the eleventh generation. Geophysical Journal International 183:1216–1230 Available: 10.1111/j.1365-246X.2010.04804.x [doi].

